# The Digital Diabetes Patient Reminder Tool for Adults With Type 2 Diabetes: Protocol for a Proof-of-Concept Randomized Controlled Trial

**DOI:** 10.2196/93006

**Published:** 2026-09-22

**Authors:** Barbara Annancy, Steve Van den Bulck, Steffen Fieuws, Laura Van den Mooter, Roman Vangoitsenhoven, Geert Goderis

**Affiliations:** 1 Academic Center for General Practice, Department of Public Health and Primary Care, KU Leuven, Kapucijnenvoer 7 blok g - bus 7001, Leuven, Flanders, 3000, Belgium, 32 16330078; 2 Research Group Healthcare and Ethics, Faculty of Medicine and Life Sciences, UHasselt, Hasselt, Belgium; 3 Leuven Biostatistics and Statistical Bioinformatics Centre (L-BioStat), Department of Public Health and Primary Care, KU Leuven, Leuven, Flanders, Belgium; 4 Endocrinology, Department of Endocrinology And Diabetology, Regional Hospital of Tienen, Tienen, Belgium; 5 Endocrinology, Department of Endocrinology, University Hospital of Leuven, Leuven, Belgium; 6 Clinical and Experimental Endocrinology, Department of Chronic diseases and Metabolism, KU Leuven, Leuven, Flanders, Belgium

**Keywords:** diabetes, type 2 diabetes mellitus, digital intervention, mobile health app, eHealth, mHealth, self-care, self-management, Joint Action for Cardiovascular Diseases and Diabetes (JACARDI), Digital Diabetes Patient Reminder (DIPAR)

## Abstract

**Background:**

About 828 million adults were estimated to be living with diabetes, and this is projected to affect 1 in 9 adults by 2045. In Belgium, 7.6% of adults have been diagnosed with the disease; however, approximately 1 in 3 people remain unaware of their condition. Advances in information and communication technology (ICT) have improved care quality and health management, and facilitated communication between health care providers and patients. The Digital Diabetes Patient Reminder (DIPAR) tool was developed as an extension of the existing patient portal to support diabetes management through automated reminders for overdue monitoring, and a consultation dashboard that facilitates communication between patients and health care providers. This pilot study was conducted within the European Joint Action on Cardiovascular Diseases and Diabetes framework.

**Objective:**

This trial aims to evaluate whether the implementation of the DIPAR tool improves adherence to recommended diabetes follow-up. The primary objective is to determine whether the intervention increases the proportion of adults with type 2 diabetes mellitus (T2DM) who receive at least one documented annual albuminuria measurement in the electronic medical records (EMR). The key secondary objective is to assess whether the intervention increases the proportion of patients with at least 2 documented hemoglobin A_1c_ (HbA_1c_) measurements within 12 months. Exploratory objectives include evaluating changes in diabetes-related clinical parameters (HbA_1c_, albuminuria, low-density lipoprotein (LDL)–cholesterol, and systolic blood pressure), as well as the usability and acceptability of the intervention among patients and health care professionals.

**Methods:**

This multicenter, pragmatic, parallel-group, proof-of-concept randomized controlled trial (RCT) will recruit approximately 200 adults with T2DM receiving outpatient diabetes care at 2 hospitals in Flanders, Belgium. Participants will be randomized (1:1), stratified only by study center, to receive either the DIPAR tool extension in addition to usual care or usual care alone using the standard patient portal. The intervention consists of automated reminders for timely follow-up and a consultation dashboard to be used by patients and physicians. The intervention will last 12 months. Clinical outcomes will be extracted from the EMR, while usability and acceptability will be evaluated using the Dutch and English versions of the Mobile Health App Usability Questionnaire (MAUQ), semistructured interviews, and focus group discussions. The primary analysis will follow the intention-to-treat principle using regression-based methods appropriate for each outcome.

**Results:**

Recruitment started in October 2025 and was completed in December 2025. A total of 201 patients were recruited, with 100 participants allocated to the intervention group. Data extraction did not commence until March 2026, after the completion of the statistical analysis plan and the protocol submission. Data analysis has not started.

**Conclusions:**

This study will provide insights into the potential added value of a digital reminder tool for T2DM management in a real-world setting as well as the usability and acceptability of this tool.

## Introduction

### Background

According to the World Health Organization (WHO), diabetes, a chronic noncommunicable disease, was the direct cause of 1.6 million deaths in 2021 [1]. Type 2 diabetes mellitus (T2DM) accounts for approximately 90%‐95% of diabetes cases, with its prevalence increasing globally, including among younger individuals [1,2]. Factors such as obesity, limited physical activity, lower socioeconomic status, ethnicity, and family history significantly contribute to the risk of developing early-onset T2DM [3,4]. Treatment for T2DM traditionally focuses on lifestyle interventions such as a healthier diet, increased physical activity, and glucose-lowering medications when necessary [5]. Recently, management strategies have shifted to a broader, holistic approach that targets not only glucose levels but also body weight, blood pressure, lipid profiles, and kidney function, addressing the comprehensive cardiometabolic health of individuals [5,6].

As of 2021, 529 million people worldwide were living with diabetes, a number projected to rise to 1.3 billion by 2050. This alarming trend highlights the global burden of diabetes [7,8]. In Belgium, T2DM diagnoses have risen from 5.4% in 2012 to 7.1% in 2022, with an estimated 1.14 million Belgians affected, including approximately 400,000 undiagnosed cases [9]. The severe complications and financial burdens associated with T2DM emphasize the critical need for strategies to halt this epidemic and improve diabetes management [10].

Advances in information and communication technology (ICT) have created opportunities to enhance health management, improve care quality, reduce medical errors, and facilitate communication between health care professionals [11-13]. Numerous studies have demonstrated the effectiveness of digital tools in supporting T2DM management. For example, Yang et al [14] conducted a randomized controlled trial (RCT) in which patients with T2DM used a mobile app for self-monitoring blood glucose and receiving physician feedback. After 3 months, the intervention group showed significant reductions in hemoglobin A_1c_ (HbA_1c_) levels compared with the control group. Similarly, Cho et al [15] reported that an internet-based device enabling interactive communication and automated health data uploads significantly reduced HbA_1c_ levels after 6 months in their intervention group. Other studies highlight that digital interventions, particularly those providing personalized feedback and reminders, can improve diabetes-related clinical outcomes (eg, HbA_1c_, BMI, and fasting glucose), enhance the management process (eg, medication adherence and follow-up visits), and boost patient motivation, knowledge, and self-management skills [16-19].

The Digital Diabetes Patient Reminder (DIPAR) tool differs from previous digital diabetes interventions in several important respects. First, this extension is embedded within an already existing patient portal (MyNexuzhealth; Nexuzhealth NV) and electronic medical record (EMR) infrastructure, allowing implementation within routine diabetes care without requiring the use of an additional stand-alone application. Second, the intervention targets both patients and health care professionals through 2 components: automated reminder notifications that promote timely diabetes monitoring and a consultation dashboard to facilitate and support communication during outpatient consultations.

This proof-of-concept trial will evaluate the effectiveness of the intervention in improving adherence to recommended diabetes monitoring and potentially generate evidence regarding the implementation of an integrated digital health intervention within routine diabetes care in a real-world setting [20]. Furthermore, this study combines the evaluation of process-of-care outcomes with implementation outcomes, including acceptability, usability, and barriers and facilitators to implementation [21].

The Joint Action on Cardiovascular Diseases and Diabetes (JACARDI) is a recent EU4Health program that aims to reduce the burden of cardiovascular disease and diabetes by uniting 21 European countries with more than 140 pilot projects that aim to address every aspect of the patient journey [22]. The DIPAR [23] pilot is one of the 143 pilot interventions being conducted in Leuven, Belgium, and falls under the JACARDI work package for self-management that focuses on various aspects of patients’ self-management, including making lifestyle changes, self-monitoring of symptoms and self-treatment, communicating with care professionals, and coping with the consequences of the disease and treatment in daily life [22-24]. The DIPAR pilot aims to develop, implement, and evaluate the DIPAR tool, which has the potential to transform diabetes care by enhancing both clinical and process outcomes. This study will evaluate the tool’s impact in a real-world clinical setting, thereby addressing a significant gap in current T2DM management strategies.

### Explanation for the Choice of Comparator

The standard of diabetes care provided to patients, as determined by their treating physician, was selected as the comparator, as it reflects the current clinical practice for the management of T2DM in the study setting. At the time of the study initiation, no digital tool comparable to DIPAR has been routinely implemented as part of the standard of care. Comparing the DIPAR intervention to usual care ensures the assessment of the effect of this intervention beyond the current clinical workflows. This will allow for the evaluation of whether the DIPAR tool improves adherence to recommended diabetes monitoring practices beyond what is currently achieved through clinical follow-up alone.

### Study Objectives and Aims

#### Primary Objective

The primary objective is to assess whether the implementation of the DIPAR tool increases the proportion of patients who receive at least one annual measurement of albuminuria, performed by either the general practitioner (GP) or their diabetes specialist, as documented in the EMR, thereby reflecting improved monitoring of diabetes-related parameters.

Annual albuminuria assessment was selected as the primary outcome because it is a key quality indicator in diabetes care, recommended by both Belgian and international clinical practice guidelines for the early detection and management of diabetic kidney disease [25-28]. Although annual albuminuria screening is recommended for all adults with type 2 diabetes, adherence to this recommendation remains suboptimal in Belgium. National quality indicators show that only 35.8% of individuals, managed predominantly in primary care, received the recommended annual albuminuria assessment [29]. Among patients receiving shared care between primary and secondary care, adherence increased from 62.4% in 2021 to 67.4% in 2024, indicating improvement but also substantial remaining scope for further optimization [30]. As the DIPAR intervention aims to improve adherence to recommended diabetes monitoring rather than directly influence clinical outcomes, albuminuria documentation was considered the most appropriate primary end point.

#### Secondary Objectives

The key secondary objectives are:

- To assess whether the proportion of patients with at least 2 documented HbA_1c_ measurements in the EMRs of the GP or diabetes specialist increases after the implementation of the DIPAR tool.

Exploratory secondary objectives are as follows:

- To determine the proportion of patients with T2DM who have their HbA_1c_ measurements documented in the EMR by the GP and/or the diabetes specialist.
- To explore the perceived usability, acceptability, and general perceptions of the digital tool among both patients and health care professionals, as well as its potential to improve self-management, self-care, and health literacy in patients within the intervention group.
- To evaluate participant engagement with the patient-facing consultation dashboard by assessing completion of the structured patient questionnaire integrated within the MyNexuzhealth platform.
- To explore whether the implementation of DIPAR is associated with changes in 6 diabetes-related clinical parameters, specifically HbA_1c_, albuminuria, cholesterol, blood pressure, estimated glomerular filtration rate (eGFR), and creatinine

## Methods

### Ethical Considerations

This study, with internal reference number S70410, obtained ethical approval from the central-acting Ethics Committee Research at the University Hospital of Leuven (UZ Leuven)/Catholic University of Leuven (KU Leuven) on June 20, 2025, and from the local Ethical Committee of Regional Hospital of Tienen (RZ Tienen) on April 10, 2025. The study will be conducted according to the principles of the Declaration of Helsinki. Data will be collected and processed in accordance with the General Data Protection Regulation (GDPR) of the European Union (EU; 2016/679).

### Trial Design

This is a 2-center study that will be conducted as an open-label, pragmatic, randomized proof-of-concept controlled trial with blinded statistical analysis. A 2-arm parallel-group design will be employed with participants randomly assigned to either the intervention or control group, comparing the usual diabetes care with the DIPAR tool. The trial is designed within a superiority framework and is exploratory in nature, as it aims to provide preliminary evidence of whether the use of the DIPAR tool can improve process-of-care outcomes.

### Patient and Public Involvement

Neither patients nor the public were engaged in the design, conducting, or reporting of this trial.

### Trial Setting

The trial will be conducted at the department of endocrinology of a large university hospital (UZ Leuven) and a smaller regional hospital (RZ Tienen) in Flemish Brabant, Flanders, Belgium.

### Eligibility Criteria for Sites and Those Delivering Interventions

As the digital intervention will be provided through an already existing patient portal developed by the Nexuzhealth software, the tool will only be made available to the participants by a member of the research team that developed the digital tool. Written informed consent from eligible participants will be obtained prior to enrollment, randomization, and any other study-related procedures. Consent will either be obtained by the local principal investigators (PIs; RV and LVdM) at each site or trained subinvestigators of the recruiting site who have been appointed by the local PI and are familiar with the study protocol. Potential participants will receive written information about the study, and participants who indicate interest in participating will receive a verbal explanation from their treating physician or a trained team member of the study team. Adequate time to consider participation will be provided if required by the participant, and all potential participants will have the opportunity to ask questions before providing consent. Participants may also provide consent through an authorized proxy or representative in accordance with the regulations of the Ethics Committee.

Biological specimens will not be collected in this study.

### Recruitment

Participants with characteristics similar to Table 1 will be recruited for this study using 2 primary methods: recruitment through health care professionals during consultations and poster/flyer dissemination. All recruitment material can be found in Multimedia Appendices 1 and 2.

**Table 1.** Characteristics of potential trial participants.

Characteristic	The people we would expect to see included
Age	Median: 68 years; however, diabetes onset is around 45 years of age, and therefore the estimated age range would be around 45‐90 years
Sex	Female and male participants will be included
Gender	—^a^
Race, ethnicity, and ancestry	Caucasian Black/African Asian Arab Latin(o) Mixed race, which will be specified by participant Other races (that are not listed) will be specified by the study participant
Socioeconomic status	Socioeconomic status will be assessed using self-reported indicators commonly applied in clinical trials. These will include highest educational attainment, employment status, marital status, and total net financial income. These variables will be collected at baseline and used to describe the study population.
Geographic location	Flemish Brabant Walloon Brabant Limburg Antwerp
Other characteristics relevant to the trial	Patient diabetes trajectory as defined in Belgium Care trajectory (zorgtraject) Convention pathway (conventie) Pretrajectory (opstarttraject) Primary language spoken by the patient Dutch French English

a Not applicable.

#### Recruitment Through Health Care Professionals

Health care professionals, including endocrinologists, GPs, diabetes educators, and nurses at the participating hospitals, will assist in identifying potentially eligible participants during routine clinical consultations. This strategy leverages the existing therapeutic relationship to facilitate the identification of eligible patients while minimizing disruption to routine clinical care.

During routine consultations, health care professionals will:

- Identify potentially eligible patients based on predefined inclusion and exclusion criteria and information available in their EMRs;
- Provide eligible patients with study information before or during their consultation, describing the study objectives, procedures, and the voluntary nature of participation; and
- Ask interested patients whether they agree to be contacted by the research team or refer them directly to the study coordinator for further discussion and enrollment.

Health care professionals will not obtain written informed consent, perform participant enrollment, or conduct randomization. All informed consent procedures, eligibility confirmation, enrollment, allocation concealment, and randomization will be performed exclusively by the research team.

#### Recruitment Through Flyer Dissemination

To support the first method of recruitment, patient recruitment will be supplemented by the distribution of study flyers and posters. These materials will be displayed in high-traffic areas within the participating hospitals, such as waiting rooms and noticeboards. If necessary, flyers will be distributed through the Diabetes Liga patient organization. All recruitment materials will be reviewed and approved by the ethics committee to ensure that they are clear, appropriate, and aligned with ethical standards.

The flyers and posters will:

- Provide a concise overview of the study, including the objectives, eligibility criteria, and potential benefits of participation;
- Include clear contact information for the study team, allowing interested individuals to contact the team directly for more information or to initiate enrollment; and
- Emphasize the voluntary nature of participation and ensure that no coercive language is used.

Patients who express interest will be referred to their treating endocrinologist to confirm eligibility and ensure they meet the inclusion criteria according to Textbox 1. Recruitment at both centers will start during the same week.

Textbox 1. **Eligibility criteria for participants.**

**Inclusion criteria**

Participants must meet all of the following criteria to be eligible for inclusion in the study:

1. Adults aged ≥18 years;
2. Treated and followed up for type 2 diabetes at the Department of Endocrinology at University Hospital of Leuven (UZ Leuven) or Regional Hospital of Tienen (RZ Tienen);
3. Have access to the MyNexuzhealth patient portal and the ability to use it;
4. Have the ability to understand Dutch, French, or English; and
5. Have provided voluntary written informed consent, or have obtained consent from their legally authorized representative, before any screening procedures.

**Exclusion criteria**:

Participants meeting any of the following criteria will be excluded from the study:

1. Pregnant or hospitalized at the time of recruitment;
2. Have a mental or cognitive impairment that interferes with their ability to use digital tools, access the MyNexuzhealth patient portal, or provide informed consent; and
3. Deemed ineligible by the treating physicians.

### Study Intervention

#### Intervention and Comparator Description

##### DIPAR Intervention Plus Usual Care

###### Overview

The DIPAR tool is an innovative extension embedded within the MyNexuzhealth patient portal [31], a widely used and approved platform in Flanders. This portal is part of the larger Nexuz platform, which integrates with the EMR system, the clinical workstation (klinisch werk station [KWS]), used by health care providers (HCPs) in several Flemish health institutions. All necessary data flows used to create and ensure the proper functioning of this tool are automatically logged within the Nexuzhealth platform. The MyNexuzhealth portal allows patients to securely and conveniently access their medical records, such as laboratory results, medical reports, images, and questionnaires. It also facilitates the scheduling and viewing of medical appointments and provides easy access to administrative and admission documents. However, documented patient records are not shared or interchangeable with electronic records provided by other medical software providers for other health care professionals. HCPs, such as GPs, however, can request patient data through a regional and federal network called the Hub [32]. This tool is provided in Dutch and French, as these are the predominant languages in Flanders, Belgium. The DIPAR intervention used in this trial corresponds to a fixed, trial-specific software version embedded within the MyNexuzhealth platform. No functional changes to the reminder logic, dashboard structure, visualization rules, or content were permitted after recruitment or during follow-up.

The DIPAR tool consists of 2 core functionalities designed to support diabetes management: a reminder function and a consultation dashboard.

###### Reminder Function

The reminder feature aims to encourage timely diabetes-related clinical follow-up in accordance with Belgian diabetes care recommendations. The DIPAR intervention is fully integrated within the MyNexuzhealth patient portal. Participants randomized to the intervention group receive automated reminder notifications when HbA_1c_ or albuminuria measurements become overdue, based on the recommended minimum monitoring intervals in Belgium (biannual HbA_1c_ and annual albuminuria measurements).

Notifications are delivered through the MyNexuzhealth platform and are generated automatically according to predefined time-based monitoring rules, which determine whether the recommended interval since the participant’s most recent documented HbA_1c_ or albuminuria measurement has been exceeded. The generation of reminders is therefore independent of the laboratory test results or their corresponding clinical values.

The method by which participants are notified (ie, a push notification through the MyNexuzhealth mobile app, email notification, or text message) depends on the communication preferences that participants had already configured within their MyNexuzhealth account before study participation. Consequently, the DIPAR intervention does not alter participants’ existing notification settings but uses their established communication preferences to maximize the likelihood that reminder notifications are received. Participants must log into the MyNexuzhealth portal (through the mobile app or webpage) to view the complete reminder message and accompanying information.

The reminder text was agreed upon by endocrinologists, GPs, and a study nurse either from the participating trial centers or directly from the research team together with the software developers. The message was provided in the language in which the patient has installed their app. The following (Table 2) is an example of the Dutch reminder text for both HbA_1c_ and albuminuria provided to Dutch patients and translated by the authors to English.

**Table 2.** Reminder message from Digital Diabetes Patient Reminder (DIPAR) for study participants.

Original Dutch text		English translation
Reminder for HbA_1c_^a^ registration		
	Onderwerp**:** Reminder: HbA_1c_ Bericht: Beste patiënt, Volgens onze informatie werd in de afgelopen 7 maanden geen gemiddelde bloedsuikerwaarde (HbA_1c_) bepaald. Voor een optimale opvolging van diabetes gebeurt dit best minstens 1 x/ 6 maanden. Gelieve contact op te nemen met uw huisarts of behandelende endocrinoloog om dit in orde te brengen, tenzij de bepaling wel recent gebeurd is, of al gepland is.	Subject: Reminder: HbA_1c_ Message: Dear patient, according to our records, no average blood glucose value (HbA_1c_) has been measured in the past 7 months. For optimal diabetes follow-up, this test should preferably be performed at least once every 6 months. Please contact your general practitioner or treating endocrinologist to arrange this, unless the test has been performed recently or is already scheduled.
Reminder for (micro) Albuminuria registration		
	Onderwerp**:** reminder: microalbuminurie Bericht: Beste patiënt, Volgens onze informatie werd in de afgelopen 13 maanden geen eiwitscreening (microalbuminurie) in de urine uitgevoerd. Voor een optimale opvolging van diabetes gebeurt dit best minstens 1x/jaar. Gelieve contact op te nemen met uw huisarts of behandelende endocrinoloog om dit in orde te brengen, tenzij de bepaling wel recent gebeurd is, of al gepland is.	Subject: reminder: microalbuminuria Message: Dear patient, according to our records, no urine protein screening (microalbuminuria) has been performed in the past 13 months. For optimal diabetes follow-up, this test should preferably be performed at least once a year. Please contact your general practitioner or treating endocrinologist to arrange this, unless the test has been performed recently or is already scheduled.

a HbA_1c_: hemoglobin A_1c_.

Reminders will be scheduled for the notifications as follows:

- For HbA_1c_:
  - First reminder after 7 months without a measurement, and
  - Second reminder 4 weeks later if still overdue.
- For albuminuria:
  - First reminder after 13 months without a measurement, and
  - Second reminder 4 weeks later if still overdue.

###### Consultation Dashboard

The consultation dashboard is the second core component of the DIPAR extension and is fully integrated within the Nexuz platform. Participants can access this via the MyNexuzhealth web portal, which is also accessible via the MyNexuzhealth mobile app. For health care professionals, the dashboard is embedded directly within the EMR system used at the participating institutions and does not require access to a separate software platform or additional login.

**Figure 1.**
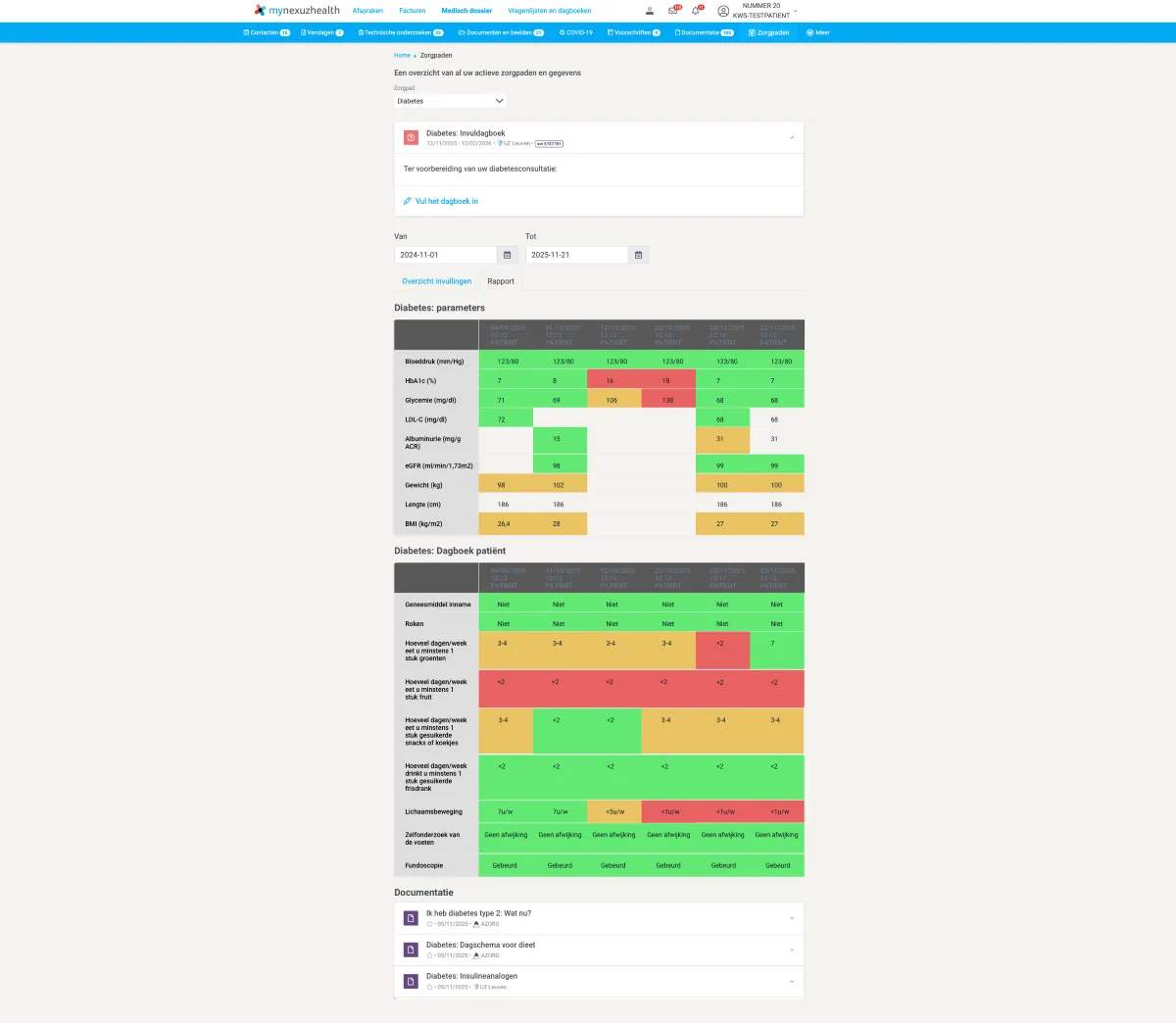
The Digital Diabetes Patient Reminder (DIPAR) color-coded dashboard for diabetes and lifestyle parameters.

This will consist of 2 complementary visualizations. The first section of dashboard (Figure 1) presents diabetes-related clinical parameters from the KWS system. These include: HbA_1c_, low-density lipoprotein (LDL-cholesterol; mg/dL), systolic blood pressure (mm Hg), albuminuria (g/g albumin-to-creatinine ratio [ACR]), and eGFR (mL/min/1.73 m^2^), routinely collected clinical data available within the EMR.

The second section of the dashboard (Figure 1) focuses on lifestyle-related information to support person-centered diabetes consultations. Lifestyle information is obtained through a structured patient questionnaire integrated within the MyNexuzhealth platform. Participants are encouraged to complete the questionnaire prior to their scheduled outpatient consultation to ensure that the information is available during the consultations, although they may update the questionnaire at any time according to their preferences. The questionnaire collects information on their medication adherence, smoking status, dietary habits, physical activity, body weight, from which BMI is automatically calculated, and completion of recommended eye and feet examinations. An additional free-text field allows participants to report relevant health information they wish to discuss with their HCPs during consultation.

Clinical and lifestyle parameters will be displayed in 4 main color codes, including green, yellow, dark orange, and gray. Parameters with values within predefined target ranges will be reflected in green, values approaching predefined thresholds will be displayed in yellow, and values outside the recommended targets will be displayed in dark orange. If no values are available in the KWS system or patient-reported questionnaire, they will be displayed in gray. The dashboard is accessible during routine outpatient consultations and provides a structured overview of overdue diabetes monitoring, thereby facilitating discussion of recommended follow-up with patients as part of standard clinical care.

Screenshots of DIPAR are provided to support reproducibility of the intervention’s core visual and functional components in other digital health settings.

Up to 48 hours prior to a consultation at the participating institution, participants will receive reminders to complete a questionnaire, from which the data regarding the lifestyle parameters will be retrieved for the lifestyle dashboard. This dashboard may be used by patients together with their physicians during the consultation to provide insights into their behavior and attitudes toward self-management during consultations. To minimize the risk of performance bias, health care professionals at both participating centers will continue to provide diabetes care according to established clinical practice and national guidelines throughout the study period. No additional training, performance targets, or changes to routine follow-up schedules will be introduced as part of the trial. The intervention is patient-directed and integrated within the MyNexuzhealth platform; therefore, clinicians caring for participants in the control group will continue to use their usual clinical workflows without access to the DIPAR functionalities. Any changes in monitoring or follow-up will remain at the discretion of the treating physician and reflect routine clinical decision-making rather than protocol-mandated procedures.

Overall, the DIPAR tool is designed to enhance patient engagement, improve adherence to follow-up schedules, and empower patients in managing their diabetes through accessible and interactive digital support. This is, however, only a supplement to the usual care, and patients will continue to receive their standard of care as planned by their HCPs. DIPAR does not replace, modify, or restrict any clinical decisions, investigations, or follow-up schedules determined by HCPs.

### Usual Care

All participants in the trial will continue to receive their usual diabetes care in accordance with Belgian national diabetes care pathways. Diabetes care in Belgium is delivered through 3 national structured pathways, namely the diabetes start trajectory, the diabetes care trajectory, and the convention pathway, with patients in the latter 2 pathways receiving part or most of their care within these settings [33,34]. The diabetes care trajectory includes individuals requiring up to 2 daily injections of insulin or other glucose-lowering therapies such as glucagon-like peptide-1 (GLP-1) receptor agonists. These patients are primarily managed by their GP, with at least one annual follow-up consultation by an endocrinologist. In contrast, individuals with complex insulin regimens, including multiple daily insulin injections (≥3 injections/day) or continuous subcutaneous glucose infusion, are managed within the diabetes convention pathway, where follow-up is coordinated by specialized endocrinology centers and includes a minimum of 2 specialists.

Both care pathways integrate multidisciplinary diabetes management across primary and specialized care and include structured consultations with GPs, endocrinologists, diabetes educators, dietitians, and podiatrists, according to national reimbursement criteria and clinical practice recommendations. Patients also have access to reimbursed diabetes technologies, including continuous glucose monitoring systems, resulting in a highly standardized approach to diabetes management across secondary care centers [35,36].

Consequently, although UZ Leuven is a university hospital and RZ Tienen is a regional hospital, both institutions function as recognized diabetes centers operating within the same national care framework, thereby minimizing intercare differences in routine diabetes care. Both RZ Tienen and UZ Leuven function as secondary care centers within this framework.

Participants in both study arms will retain their standard access to the MyNexuzhealth patient portal, which enables patients to view their electronic health records, laboratory results, appointments, and hospital invoices.

### Criteria for Discontinuing or Modifying Allocated Intervention/Comparator

As the DIPAR intervention is a low-risk digital health intervention, no protocol-defined clinical criteria require discontinuation or modification of the allocated intervention. Participants may discontinue use of the intervention while remaining in the study under the following circumstances: (1) persistent technical difficulties that cannot be resolved despite reasonable efforts by the research team and software developers, thereby preventing continued use of the DIPAR tool; or (2) the participant voluntarily chooses to discontinue the use of the intervention but agrees to continue study participation. The reason for discontinuation will be documented in all cases. Discontinuation of the intervention will not constitute a protocol deviation requiring exclusion from the primary analysis. All randomized participants will be analyzed according to the intention-to-treat (ITT) principle irrespective of intervention discontinuation, technical difficulties, or adherence.

### Strategies to Improve Adherence to Intervention/Comparator

**Figure 2.**
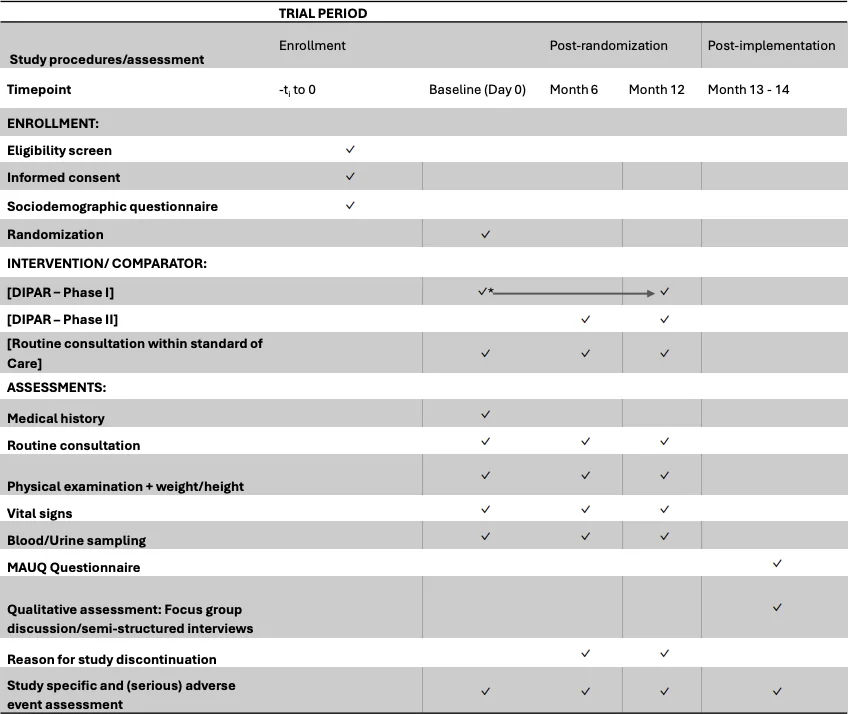
Overview of the participant timeline. *Accessibility to the DIPAR tool after 48 hours of randomization. DIPAR: Digital Diabetes Patient Reminder; MAUQ: Mobile Health App Usability Questionnaire.

As the digital tool used within this trial includes an automated reminder system that sends notifications to participants at predefined intervals (including reminders every 6 months and reminders before scheduled appointments with endocrinologists), adherence to the intervention will be supported in this manner. Health care professionals will also be encouraged to refer and use the application with the patient during routine consultations to reinforce its use, without providing additional interventions beyond the usual standard of care. No other specific adherence-enhancing strategies will be applied to the comparator group beyond the routine clinical practice. Figure 2 represents a general overview of the participant timeline during the DIPAR study.

As this is a digital intervention that does not aim to interfere with the patient’s clinical routine, no concomitant care or interventions are prohibited during the trial. However, participants may be advised against participating in clinical trials with a digital intervention and a similar study objective to that of this trial.

Irrespective of the allocation group, all participants will continue to receive standard diabetes care according to routine clinical practice. Given the minimal-risk nature of the DIPAR digital intervention, this study will not involve additional ancillary care or specific posttrial care beyond the usual care.

In the event of harm related to trial participation, participants will be covered according to standard clinical procedures and covered by the sponsor’s clinical trial insurance in line with institutional and national regulations. Participants will not receive any financial compensation for participation in the study. Upon trial completion, all participants will have access to the DIPAR digital intervention.

### Outcomes

All outcomes for the DIPAR study objectives can be found in Multimedia Appendix 3, with their measurement variable, analysis metric, method of aggregation, and time point.

### Translated Dutch Mobile Health App Usability Questionnaire Scale

This Mobile Health App Usability Questionnaire (MAUQ) is a widely used and validated instrument for assessing user feedback on medical digital tools, specifically evaluating their ease of use, satisfaction, and perceived usefulness [37]. It has demonstrated strong reliability and validity in measuring mobile health (mHealth) app usability. The Dutch version of the MAUQ, which was translated and validated by van de Weerd et al [38], or the English version, will be administered to participants in the intervention group at month 14 of the study using REDCap (Vanderbilt University) [39]. For this study, the original 21-item questionnaire has been adapted to 16 questions, focusing on aspects most relevant to the DIPAR extension. Completing the questionnaire is expected to take approximately 10 minutes, with responses recorded on a 5-point Likert scale ranging from 1=“Not at all” (“Sterk mee oneens”) to 5=“Strongly agree” (“Sterk mee eens”). The mean and SD for each of the 3 domains and the total usability score will be calculated, according to the MAUQ instruction guide [37]. Both the English and Dutch versions of the questionnaires can be found in the Multimedia Appendix 1.

### Qualitative Focus Group Discussions and Semistructured Interviews

To complement the qualitative usability assessment, qualitative substudies with focus group discussions and/or semistructured interviews will be conducted with participants and HCPs to gain a deeper understanding of their experiences with the DIPAR intervention. This will specifically explore the participants’ perceptions of the usability, acceptability, and perceived usefulness of the DIPAR intervention; facilitators and barriers to its routine use and implementation; its perceived influence on diabetes self-management, communication, and clinical consultations; and suggestions for improving the functionality, design, and future implementation of the intervention.

### Harms

#### Adverse Events

An adverse event (AE) is any untoward medical occurrence in a patient or participant during an intervention that does not necessarily have a causal relationship with this treatment.

An AE can therefore be any unfavorable and unintended sign (including an abnormal laboratory finding), symptom, or disease temporally associated with the use of a product, whether or not considered related to the product. Any worsening (ie, any clinically significant adverse change in the frequency or intensity of a preexisting condition) should be considered an AE.

#### Adverse Reaction

An adverse reaction (AR) is any untoward and unintended response to an investigational medicinal product or to an intervention and, when an investigational product is concerned, related to any dose administered.

#### Serious Adverse Event

##### Overview

A serious adverse event (SAE) is any untoward medical occurrence that results in any of the following:

- Death
- A life-threatening event, in the definition of an SAE, this refers to an event in which the participant was at risk of death at the time of the event. It does not refer to an event that hypothetically might have caused death if it was a more severe experience
- Inpatient hospitalization or prolongation of existing hospitalization
- A persistent or significant disability or incapacity
- A congenital anomaly or birth defect
- Important medical events that may be considered an SAE when—based on appropriate medical judgment—may jeopardize the participant and may require medical or surgical intervention to prevent one of the above outcomes

Any elective hospitalizations will not be classified as an SAE.

The risks associated with the study-specific intervention primarily involve technical issues, user challenges, psychological impacts, and privacy concerns.

##### Technical Issues and Reliability

As a digital tool, the intervention may experience technical glitches that disrupt access or functionality. The tool may also have compatibility issues across different devices, limiting accessibility for some participants.

##### User Challenges

Participants may have varying levels of digital literacy, making it difficult for them to fully engage with the tool. Some may experience frustration or disengagement, leading to noncompliance with reminders or abandonment of the DIPAR extension altogether.

##### Privacy and Personal Information

As part of the study, participants will be asked to share personal information during semistructured interviews and focus group discussions. If this data are not properly handled, it could lead to breaches of privacy and misuse of personal health information.

All AEs will be documented promptly upon identification. This study is categorized as low risk. Patients will not be required to undertake any other forms of laboratory assessment or evaluation besides the required routine follow-up care that is specific to this study. Study-specific AEs (SSAEs) as well as the study end points will be excluded from expedited reporting, but these will be documented in the electronic case report form (eCRF) and reported to the study sponsor. If there are study-specific AEs, these will also be recorded and reported in subsequent trial publications.

Investigators will seek information on AEs during each patient contact. All events, whether reported by the patient or noted by study staff, will be recorded in the patient’s medical record and in the eCRF within a reasonable time after becoming aware of the event. If available, the diagnosis should be reported on the AE page, rather than the individual signs or symptoms. If no diagnosis is available, the investigator should record each sign and symptom as individual AEs.

The following information will be recorded for each AE:

- AE description
- Start and stop date of the AE
- Severity
- Seriousness
- Causality assessment of the study interventions

As this is a low-risk trial, no safety information will be included in the annual progress report submitted to the coordinating ethics committee.

### Death

All deaths will be reported without delay to the sponsor (irrespective of whether the death is related to disease progression, study procedure, or is an unrelated event). The sponsor will notify all deaths, as soon as possible after becoming aware, to the Central Ethics Committee (EC) and the EC of the concerned site and provide additional information if requested.

### Participant Timeline

#### Sample Size

The sample size was calculated using the operational definition of the primary end point, namely the presence of at least one albuminuria measurement documented in the EMR within a 12-month period. Based on data collected from the Department of Endocrinology at the UZ Leuven, between October 2023 and October 2024, albuminuria measurement and documentation in the EMR documented by the endocrinologist was observed in 1600 out of 2800 patients with T2DM, corresponding to a baseline proportion of 57%. The study hypothesizes that the implementation of the DIPAR intervention could increase this proportion from 57% to 77%, corresponding to an absolute improvement of 20 percentage points.

This anticipated effect was not derived from previous randomized trials, as comparable integrated portal interventions targeting albuminuria monitoring are currently lacking. Rather, the expected improvement was determined a priori through discussion within the multidisciplinary research team and was considered both clinically meaningful and feasible for a patient-targeted digital reminder intervention targeting a process-of-care outcome. An absolute increase of 20 percentage points was considered clinically meaningful and achievable for, and sufficiently large to justify future scale-up and implementation if the intervention demonstrated effectiveness.

The required sample size was calculated using a 2-sided chi-square test for comparison of proportions, with a significance level α=.05 and 80% power to detect this difference between the intervention and control groups. Under these assumptions, a total of 172 participants (86 per group) is required.

The study aimed to recruit a total sample size of 200 participants. Although loss to follow-up is expected to be limited, as the intervention does not alter standard clinical care or follow-up procedures, the target sample size was inflated to account for an anticipated dropout rate slightly lower than 15%, particularly to ensure sufficient data for exploratory end points.

Due to the differences in recruitment capacity across the participating centers, participants will not be enrolled in equal numbers at each site. The UZ Leuven, a larger tertiary university hospital with extensive experience in clinical research, will recruit approximately two-thirds of the total sample (n=140). The remaining participants (n=60) will be recruited at the regional hospital RZ Tienen. Recruitment will continue until the target sample size is achieved and the required number of participants at each site is reached. If the required target size is reached at UZ Leuven before RZ Tienen, recruitment will continue until exactly 60 participants are recruited at Tienen.

#### Randomization

The random allocation sequence will be generated by an independent member of the research team who will not be directly involved in the study, including the participant recruitment and outcome assessment. Randomization will be performed using a computer-generated random sequence.

Participants will be randomized using a 1:1 allocation ratio between the intervention and control groups. The computer-generated allocation sequence will use permuted blocks of varying sizes to maintain the balance between study arms.

Randomization will be stratified by participating hospitals to ensure balanced representation of participants from each participating site across the intervention and control study arms.

Allocation concealment of the computer-generated randomization sequence will be ensured using the REDCap [39] system, which will function as an Interactive Web Response System (IWRS). This system will ensure secure implementation of randomization, minimize allocation bias, reduce the risk of manual errors, and maintain an auditable record of randomization details. After a participant has been confirmed as eligible and has provided written informed consent, the recruiting investigator will request group allocation through REDCap. The assigned study arm will be revealed only at the time of allocation, thereby ensuring that the randomization sequence remains concealed until interventions are assigned. Randomization will be performed by a designated study team member within the REDCap system, who will be involved in participant enrollment but not outcome assessment of the study and will not have access to the random allocation sequence, which will remain accessible only to the independent team member responsible for generating and managing the sequence.

### Assignment of Interventions: Blinding

As this is a digital intervention, whereby the DIPAR tool will only be available for the intervention group, concealment of the allocation from the participants and outcome assessors will not be possible. However, the statistical data analyst of this study will be blinded to the group allocation. Statistical analyses will be conducted using a treatment-coded dataset in which the treatment groups are labeled with neutral identifiers (eg, coded as Group A and Group B). The statistician will remain unaware of which code corresponds to the intervention or control group until the primary analysis has been completed and the statistical analysis report has been finalized.

Furthermore, the use of objective, routinely collected electronic health records for the primary and key secondary outcomes minimizes the risk of detection and assessment bias.

There is no procedure for unblinding, as patients, investigators, and outcome assessors are aware of the group allocation.

### Data Collection and Management

All participants enrolled in this study will be assessed during routine consultations at the Department of Endocrinology in the participating institution to confirm eligibility according to the predefined inclusion and exclusion criteria.

Following informed consent and randomization, baseline data will be obtained as part of routine clinical care and will include sociodemographic characteristics, medical history, clinical parameters, medication use, and comorbidities. Sociodemographic information will be collected using a study-specific questionnaire, while clinical data will be extracted from participants’ EMRs. No additional physical examinations or laboratory assessments will be performed that deviate from standard diabetes care.

Clinical outcomes will be extracted from the EMRs at baseline, 6 months, and 12 months. SSAEs and SAEs will be collected as soon as the team becomes aware of the occurrence. Only participants allocated to the intervention group will be contacted by mail or telephone regarding SSAEs by members of the research team.

To ensure complete ascertainment of the primary and secondary process outcomes, laboratory measurements performed in both primary care (by the GPs) and secondary care (by the diabetes specialists) will be included. Laboratory results generated within the participating hospitals are automatically recorded in the hospital EMR. Measurements requested by GPs are routinely transferred to Nexuz through established clinical workflows within the regional KWS health care network. In addition, the Nexuz platform, on which the DIPAR extension is embedded, provides access to externally documented laboratory results available through its integrated health care information infrastructure. Consequently, albuminuria and HbA_1c_ measurements performed in primary care or external laboratories can be identified and retrieved for study purposes.

For each participant, all eligible albuminuria and HbA_1c_ measurements performed during the 12 months preceding study enrollment and throughout the 12-month follow-up period will be extracted. Retrieved laboratory records will be cross-checked against the hospital EMR to verify completeness and prevent duplicate counting. Where duplicate records of the same laboratory measurement are identified across different sources, they will be counted only once. All eligible laboratory measurements documented within the predefined observation periods, irrespective of whether they originated from primary or secondary care, will contribute to the assessment of the primary and secondary process-of-care outcomes.

All study data obtained from hospital records, GP documentation, questionnaires, and safety assessments will be entered into the eCRF within the REDCap [39] system hosted by UZ Leuven/KU Leuven. At 12 months, participants in the intervention group will be invited to complete the English or Dutch version of the MAUQ, either electronically via email or by telephone with a member of the research team. A purposive sample of intervention participants will subsequently be invited to participate in semistructured interviews or focus group discussions to evaluate the usability, acceptability, and implementation of the DIPAR intervention. Informed consent forms and the baseline sociodemographic questionnaire collected on paper documents will subsequently be uploaded to REDCap, and paper documents will be stored in a locked cabinet in the office of the coordinating PI (GG).

The core research team has received training in the use of the REDCap system and has role-based, password-protected access. To ensure data quality and consistency, data entry into the eCRFs will be performed by 2 designated personnel. In accordance with the GDPR, Belgian regulations, and UZ Leuven policies, all research data will be stored for at least 25 years after the end of this trial [40].

Participants in the intervention arm will be reminded of their participation in the study through the reminder function of the DIPAR tool and when they are contacted by the research team to discuss SSAEs. No reimbursements will be given to participants in the study, but participants who participate in the qualitative research will receive a gift voucher of no more than €50 (€1=US $1.16 as of September 3, 2026).

For all participants, every effort will be made to collect the outcomes and safety end points listed above.

### Confidentiality

No personal data or identifier will be uploaded to REDCap. To enable pseudonymization, participants’ names will be replaced by a 6-digit code referred to as the study ID. The names and date of birth that are assigned to a specific code will be put in a different document, a master file, and saved on the secured KU Leuven server. The other data that will be collected will also be stored separately in the eCRF. Therefore, allowing a process of pseudonymization. At months 6 and 12, when follow-up phone calls are conducted for the SSAEs, the master file will be accessed by the 2 designated personnel. The informed consent form only contains the participant’s name and signature. These forms will be stored under lock and key in the coordinating PI’s office.

### Statistical Methods and Data Analysis

#### General Statistical Principles

All statistical analyses will be conducted according to a prespecified statistical analysis plan finalized prior to database lock and before any comparative analyses of trial outcomes are undertaken. Statistical analyses will follow the ITT principle unless otherwise specified. All statistical tests will be 2-sided with a significance level of 0.05. Treatment effects will be presented together with 95% CIs. Given the proof-of-concept nature of this trial, emphasis will be placed on estimation of treatment effects and precision of estimates rather than statistical significance alone.

#### Analysis of Populations (ITT and Per Protocol)

The full analysis set (FAS) will, in accordance with the ITT principle, include all randomized patients according to their randomized treatment assignment. The FAS will be used for the evaluation of all efficacy and safety end points.

Patients from the FAS with major protocol deviations will be excluded from the per protocol set (PPS). The PPS will be reviewed and finalized prior to database lock at a blind review meeting where all attendees will be kept blind to the randomized study treatment. The blind review meeting will be attended by the PI, study statistician, and other relevant study personnel. All decisions taken at this meeting will be fully documented in a blind review document.

However, analyses of the acceptability and usability analyses will be restricted to participants in the intervention group and HCPs who provide consent to participate in the questionnaire, interviews, or focus group.

#### Descriptive Analyses

All baseline characteristics will be presented using descriptive statistics. Baseline demographic and clinical characteristics will be summarized by randomized treatment groups. Continuous variables will be presented as means (SDs) or medians (IQRs), depending on their distribution. Categorical variables will be summarized as frequencies and percentages. No formal statistical testing of baseline differences will be performed.

#### Primary Outcome

The primary end point, documentation of at least one albuminuria measurement during follow-up, will be analyzed using multivariable logistic regression. The model will include treatment trajectories (pretrajectory, care trajectory, or convention pathway), treatment allocation, and study center (UZ Leuven and RZ Tienen) as fixed effects. Results will be reported as adjusted odds ratios with corresponding 95% CIs and 2-sided *P* values. To avoid estimation problems due to quasi-separation, Firth penalized logistic regression will be used.

#### Key Secondary Outcome

The proportion of participants with at least 2 documented HbA_1c_ measurements will be analyzed using the same approach as that used for the primary outcome. The sample size was not powered for these outcomes.

#### Exploratory Secondary Outcome

The number of HbA_1c_ measurements that occurred will be assessed using a negative binomial regression model, with the allocated group and study center as fixed effects, and reported with incidence rate ratios (IRRs). For continuous secondary clinical outcomes, between-group comparisons at 6 and 12 months will be performed using linear mixed effects models, assuming that missing data are missing at random (MAR). Treatment trajectory, trial center, and study group will be included as fixed effects. Participant engagement with the consultation dashboard will be summarized descriptively. The total number of questionnaire entries and the number and proportion of participants completing at least one structured questionnaire during the study period will be reported.

Exploratory within-group analyses at baseline, 6 months, and 12 months will be derived from the same model. In all analyses, statistical uncertainty will be quantified using 95% CIs.

The study is powered to detect differences in the primary process-of-care outcome only. Analyses of clinical outcomes, including HbA_1c_, LDL cholesterol, systolic blood pressure, and albuminuria values, are exploratory and are intended to generate estimates of effect sizes and variability to inform the design and sample size calculations of a future definitive RCT. Statistical significance for these outcomes will therefore be interpreted cautiously.

#### Missing Data

Multiple imputation will be considered in the unlikely case of missing data due to consent withdrawal. In line with a treatment policy estimand, deceased patients without documentation will be counted as not meeting the end point. A sensitivity analysis restricted to participants who survived through follow-up will be performed.

If more than 10% of observations are missing for any exploratory outcome, multiple imputation by chained equations will be considered as part of a sensitivity analysis.

For the MAUQ, subscale scores will be calculated when at least 50% of the items within a subscale have been completed. Otherwise, the corresponding subscale score will be treated as missing, and no item-level imputation will be performed, as this questionnaire will only be administered once to the patients after the follow-up period.

#### Moderator Analyses

Exploratory subgroup analyses may be performed according to sex, age, study center, and diabetes treatment pathway, as defined at the baseline. For this purpose, the analysis model will be extended with the appropriate interaction terms. If needed, restricted cubic splines will be considered to allow nonlinearity in the effect of age.

#### Multiplicity

Only the primary and key secondary end points will be considered confirmatory. Analysis of secondary and exploratory outcomes will be interpreted as exploratory and hypothesis-generating. Therefore, no formal adjustment for multiplicity will be applied. Estimates will be presented together with 95% CIs to facilitate interpretation.

#### Software

All statistical analyses will be performed using R (Statistical Computing and Graphics; version 4.6.1 or later; R Foundation for Statistical Computing), SPSS Statistics (version 32.0 or later; IBM Corp), and GraphPad Prism (version 11.0.0 or later; GraphPad Software, LLC).

### Additional Considerations

#### Qualitative Analyses

Score calculations from the MAUQ will be summarized using descriptive statistics. The qualitative studies will be performed until thematic richness is obtained.

No interim analyses of efficacy are planned because outcome assessments coincide with routine clinical follow-up and the trial is not designed for early stopping.

The study protocol, statistical analysis plan, and deidentified data supporting the primary publication will be made available upon reasonable request following publication of the primary study results. Statistical code may also be shared upon reasonable request or deposited in a publicly accessible repository.

#### Trial Status

The study protocol and preliminary statistical analysis plan were developed in February 2025 and finalized in May 2025. The trial was submitted to ClinicalTrials.gov on September 24, 2025, prior to recruitment. Following the standard ClinicalTrials.gov quality-control review process, revisions to the registration record were requested to improve the specification of the registered outcome measures. These revisions were completed and resubmitted on October 7, 2025. The trial record subsequently met ClinicalTrials.gov quality control criteria on November 26, 2025, and was publicly posted on December 2, 2025 (ClinicalTrials.gov identifier: NCT07257822).

Participant recruitment commenced on October 6, 2025, and was completed on December 31, 2025, with 201 participants enrolled. Baseline data extraction commenced in March 2026, after submission of this protocol manuscript. At the time of submission of this protocol manuscript, no outcome analyses or interim analyses had been conducted. Analysis of the baseline characteristics will begin following publication of this protocol. Submission of the protocol manuscript was delayed due to awaiting the final technical description of the DIPAR dashboard for phase 2 from the software developers and the end-of-year holiday period. Although this protocol manuscript was submitted after completion of participant recruitment, all study procedures, eligibility criteria, intervention specifications, primary and secondary outcome definitions, case report forms, the statistical analysis plan, and the overall study design were finalized before recruitment commenced and remained unchanged throughout recruitment.

### Oversight and Monitoring

The UZ Leuven will act as the coordinating center of this trial. The trial steering committee will comprise the coordinating PI (GG), 2 local PIs (RV and LVdM) from each trial center, the study coordinator, and an experienced researcher. The study coordinator, together with the study nurse, is responsible for conducting the trial on a day-to-day basis and reporting any deviations to the members of the steering committee. The committee will have monthly meetings to discuss all the progress and delays and discuss eventual modifications. As this is a low-risk exploratory trial that will be closely monitored by the investigators, no data monitoring committee will be appointed.

The trial will be monitored by the study coordinator and documented in the trial logbook. This will include the inclusion rate, the rate at which participants are recruited, the reasons for declining participation, and the collection of all paper-based signed informed consent files and sociodemographic questionnaires. No additional trial audit will be performed. Any important modifications or amendments, including changes to study objectives, eligibility criteria, outcomes, study procedures, or analyses, will be formally documented as protocol amendments.

Prior to implementation, protocol amendments will be submitted for approval to the central acting EC, the research EC UZ/KU Leuven. After approval is granted by the EC, this will be updated on the trial registry of ClinicalTrials.gov where the study is published.

All relevant stakeholders, including investigators and study personnel, will be informed of approved amendments in a timely manner. If protocol modifications affect participants’ involvement or rights, participants will be informed accordingly, and revised informed consent will be obtained when required.

Minor administrative changes that do not affect participant safety, study conduct, or scientific integrity will be documented but may not require formal ethical approval, in line with local regulations.

## Results

Recruitment for the DIPAR proof-of-concept RCT commenced in October 2025 and was completed in December 2025. A total of 201 participants were enrolled across the 2 participating study sites and randomized in a 1:1 ratio to the intervention group (n=100) or the control group (n=101).

Sociodemographic questionnaires were collected during enrollment, while baseline clinical data were extracted from the participants’ EMRs in March 2026, after submission of the protocol manuscript.

The 6-month follow-up assessments are scheduled between April and June 2026, during which participants in the intervention group will also receive access to the DIPAR consultation dashboard. Clinical outcomes will be extracted from the EMR, and SSAEs related to the intervention will be assessed.

The final 12-month follow-up assessments are planned between October and December 2026, after which data collection will be completed. During this phase, participants in the intervention group will complete the English or Dutch version of the MAUQ, and a purposive sample of patients and health care professionals will be invited to participate in semistructured interviews and focus group discussions to evaluate the usability, acceptability, and implementation of the DIPAR intervention.

Following completion of data collection by the end of March 2027, quantitative and qualitative analyses will be conducted according to the prespecified statistical and qualitative analysis plans. Study findings are expected to be submitted for publication toward the end of 2027.

This trial received funding under the JACARDI project from the EU4Health Program 2021‐2027 under Grant Agreement 101126953 and was prospectively registered on ClinicalTrials.gov (NCT07257822). Trial status, including recruitment completion, study completion, and summary results, will be updated in the registry in accordance with applicable reporting requirements.

Participants will be informed of the overall study results through a summary written for a lay audience and made available upon completion of the DIPAR study. No individual participant-level results or data will be disclosed.

## Discussion

### Principal Findings

This study describes the protocol for evaluating the DIPAR tool, a digital intervention designed to support diabetes monitoring, self-management, and care processes for people with T2DM in a real-world clinical setting in Flanders, Belgium.

### Strengths

A key strength of this study is its focus on process-of-care outcomes, particularly adherence to the Belgian recommended monitoring practices of albuminuria and HbA_1c_ measurements for individuals with T2DM. Audit and feedback are frequently used to feed data back to HCPs, whereas the DIPAR tool aims to target patients through its reminder function and dashboard feature. Several studies, as discussed in the Introduction, have highlighted the benefits of personalized feedback and reminders in improving diabetes-related clinical outcomes. The outcomes in this study are clinically relevant, will be efficiently measured using routinely collected data, and are closely aligned with quality-of-care indicators in diabetes management.

Using standard of care as the comparator in this trial will allow the evaluation of DIPAR within existing clinical workflows, thereby enhancing the pragmatic nature of the study. This approach enables an assessment of whether the digital intervention provides added value beyond the current state, which could be valuable in informing future implementation decisions. Moreover, embedding the intervention within routine care reflects real-world conditions and supports the external validity of the findings, as no comparable digital reminder and dashboard tool has previously been developed or evaluated specifically for the Flemish population.

An additional strength of the study is the inclusion of an objective measure of participant engagement with the patient-facing consultation dashboard, which will support the interpretation of the intervention’s effectiveness. However, comprehensive audit data on reminder views and health care professional dashboard access are currently unavailable within the existing platform infrastructure.

Finally, this study is conducted within the JACARDI framework and therefore includes the evaluation of acceptability and usability assessments among both patients and health care professionals, which constitutes a notable strength. By incorporating qualitative and questionnaire-based evaluations, this study aims to generate insights into how DIPAR is experienced in practice and how it may support self-management, health literacy, and patient–provider communication from the perspective of its users. Finally, while changes in clinical parameters are explored, the study is not primarily powered to detect long-term clinical outcomes, and these findings should be interpreted accordingly.

### Limitations

Several limitations, however, should be considered. First, data collection will rely on routinely collected data in the EMR, which may result in missing or incomplete information, potentially affecting outcome assessment. Second, as a pilot study conducted in a specific regional context, the generalizability of the findings may be limited. Furthermore, this trial will last for only 12 months, which will not allow exploration of long-term clinical outcomes, and the findings from this study will therefore be interpreted accordingly.

A limitation of this protocol publication is that the manuscript was submitted after participant recruitment had been completed. This timing reflects the publication process rather than any changes to the study methodology. Importantly, the intervention, eligibility criteria, outcome definitions, and planned statistical analyses were finalized before recruitment commenced and remained unchanged throughout participant enrollment. No outcome data were accessed, analyzed, or used to inform modifications prior to the finalization of this protocol and statistical analysis plan manuscript, thereby minimizing the risk of analysis bias and selective outcome reporting. Publication of the protocol therefore provides a transparent, prespecified description of the study methodology to support the interpretation and reporting of the trial results.

## Supplementary material

10.2196/93006 Multimedia Appendix 1 Ethical approval and questionnaires.

10.2196/93006 Multimedia Appendix 2 Recruitment material.

10.2196/93006 Multimedia Appendix 3 Study outcomes, measurement variables, analysis metrics, and assessment time points.

## Abbreviations

ACR: albumin-to-creatinine ratio
AE: adverse event
AR: adverse reaction
DIPAR: Digital Diabetes Patient Reminder
EC: Ethics Committee
eCRF: electronic case report form
eGFR: estimated glomerular filtration rate
EMR: electronic medical record
EU: European Union
FAS: full analysis set
GDPR: General Data Protection Regulation
GLP: glucagon-like peptide-1
GP: general practitioner
HbA_1c_: hemoglobin A_1c_
HCP: health care provider
ICT: information and communication technology
IRR: incidence rate ratio
ITT: intention-to-treat
IWRS: Interactive Web Response System
JACARDI: Joint Action on Cardiovascular Diseases and Diabetes
KU Leuven: Catholic University of Leuven
KWS: klinisch werk station (clinical working station)
LDL: low-density lipoprotein
MAR: missing at random
MAUQ: Mobile Health App Usability Questionnaire
mHealth: mobile health
PI: principal investigator
PPS: per protocol set
RCT: randomized controlled trial
RZ Tienen: Regional Hospital of Tienen
SAE: serious adverse event
SSAE: study-specific adverse event
T2DM: type 2 diabetes mellitus
UZ Leuven: University Hospital of Leuven
WHO: World Health Organization
