# Supplementary material for: The Digital Diabetes Patient Reminder Tool for Adults With Type 2 Diabetes: Protocol for a Proof-of-Concept Randomized Controlled Trial
Source: JMIR Res Protoc. 2026 Sep 22;15:e93006. doi: 10.2196/93006 (PMC13597014; doi:10.2196/93006)
Supplement: Supplementary file 1 [file resprot-v15-e93006-s001.docx]

Multimedia Appendix

Contents

[Approved letter from Ethics Committee	2](#_Toc221273810)

[Central EC approval	2](#_Toc221273811)

[Local ethics committee approval	7](#_Toc221273812)

[Informed consent	9](#_Toc221273813)

[Baseline sociodemographic questionnaire	21](#_Toc221273814)

[The mHealth App Usability Questionnaire (MAUQ)	24](#_Toc221273815)

[The English version	24](#_Toc221273816)

[The Dutch translated and validated version of the MAUQ questionnaire	26](#_Toc221273817)

[References	29](#_Toc221273818)

# Approved letter from Ethics Committee

### Central EC approval

This illustrates the Ethical approval the DIPAR RCT which was obtained from the central-acting ethics committee research UZ/KU Leuven on June 20^th^ 2025.

### Local ethics committee approval

This represent the approval from the local ethics committee, obtained on April 11^th^ 2025 form the ethical committee of RZ Tienen

# Informed consent

These screenshots are copies of the 12 page English version of the informed consent file that all patients had to sign as part of the eligibility criteria for the DIPAR study. This questionnaire was provided in Dutch and French and all versions were approved by the ethical committee.

# Baseline sociodemographic questionnaire

This shows the copies of the English version of the sociodemographic questionnaire that patients completed after being deemed eligible and signing the informed consent. This questionnaire was provided in Dutch and French and all versions were approved by the ethical committee.

# The mHealth App Usability Questionnaire (MAUQ)

### The English version

These screenshots are from the mHealth App Usability Questionnaire (MAUQ) that will be used for the DIPAR tool evaluation post implementation. MAUQ is a widely used and validated instrument for assessing user feedback on medical digital tools, specifically evaluating their ease of use, satisfaction, and perceived usefulness (1). English speaking patients in the intervention group will be provided with this copy. This document was also submitted together with the study protocol and approved by the ethics committee.

### The Dutch translated and validated version of the MAUQ questionnaire

These represent the Dutch version of the MAUQ, which was translated and validated by van de Weerd, Ebbers et al. (2023) (2). Dutch speaking patients in the intervention group will be provided with this copy. This document was also submitted together with the study protocol and approved by the ethics committee.

### References

1.	Zhou L, Bao J, Setiawan IMA, Saptono A, Parmanto B. The mHealth App Usability Questionnaire (MAUQ): Development and Validation Study. JMIR Mhealth Uhealth [Internet]. 2019 [cited 2025 Feb 24];7(4):e11500. Available from: https://pmc.ncbi.nlm.nih.gov/articles/PMC6482399/

2.	van de Weerd C, Ebbers T, Smilde DEM, van Tol-Geerdink JJ, Takes RP, van den Broek GB, et al. Evaluation of a remote monitoring app in head and neck cancer follow-up care. Cancer Med [Internet]. 2023 Jul 1 [cited 2025 Feb 24];12(14):15552–66. Available from: https://onlinelibrary.wiley.com/doi/full/10.1002/cam4.6202
