# Supplementary material for: The Digital Diabetes Patient Reminder Tool for Adults With Type 2 Diabetes: Protocol for a Proof-of-Concept Randomized Controlled Trial
Source: JMIR Res Protoc. 2026 Sep 22;15:e93006. doi: 10.2196/93006 (PMC13597014; doi:10.2196/93006)
Supplement: Supplementary file 2 [file resprot-v15-e93006-s002.docx]

Multimedia Appendix 3

Contents

[Study poster	2](#_Toc221275047)

[Digital study information slide	3](#_Toc221275048)

[The DIPAR clinical trial information flyer	4](#_Toc221275049)

# Study poster

This figure shows the study poster that was made available in the waiting rooms of endocrinologists of the participating institution and was only provided in Dutch. This document was approved by the EC.

# Digital study information slide

This figure shows the digital poster that was displayed on tv screens in the waiting rooms of endocrinologists of the participating institution and was only provided in Dutch. This document was approved by the EC.

# The DIPAR clinical trial information flyer

This figure represents the one page flyer provided to patients with a brief overview of the study information and the eligibility criteria. The flyer was also provided in Dutch and French, and all documents were approved by the EC
