# Supplementary material for: The Digital Diabetes Patient Reminder Tool for Adults With Type 2 Diabetes: Protocol for a Proof-of-Concept Randomized Controlled Trial
Source: JMIR Res Protoc. 2026 Sep 22;15:e93006. doi: 10.2196/93006 (PMC13597014; doi:10.2196/93006)
Supplement: Supplementary file 3 [file resprot-v15-e93006-s003.docx]

| **Study outcomes** | **Outcome title** | **Measurement variable** | **Analysis metric** | **Method of aggregation** | **Time point** |
| --- | --- | --- | --- | --- | --- |
| Primary outcome | Documentation of albuminuria monitoring | Presence of at least one albuminuria measurement in the EMR by either a general practitioner or a diabetes specialist | Binary outcome (yes/no) indicating whether ≥1 albuminuria measurement was conducted and documented during the follow-up period | Proportion of patients meeting the criterion in intervention group compared with the control group | Assessed at 12 months post-implementation |
| Key Secondary outcome | Documentation of HbA1c monitoring | The documentation of at least 2 HbA1c measurements documented in the EMR by either a general practitioner or diabetes specialist | Binary outcome (yes/no) indicating whether ≥2 HbA1c measurements were conducted and documented during follow-up period | Proportion of patients meeting criterion in intervention group compared to the control group | Assessed at 12 months post-implementation |
| Exploratory Secondary outcomes | Documentation of HbA1c monitoring | Presence of at least one HbA1c value documented in the EMR | Binary outcome (yes/no) | Proportion of patients with ≥1 documented HbA1c measurement in the intervention compared to the control group | Assessed at 12 months-post implementation |
|  | Changes in clinical parameters from baseline to follow-up | Changes in HbA1c | Change from baseline | Based on the distribution of the changes of these clinical parameters, reporting the appropriate measure: mean ± SD and median (IQR) will then be decided.  Although these outcomes are exploratory, multiple imputations will be applied to handle missing data, and results will be summarized using medians where appropriate. | Baseline, 6 months and 12 months |
|  |  | Changes in Albuminuria | Change from baseline |  | Baseline, 6 months and 12 months |
|  |  | Changes in Creatinine | Change from baseline |  | Baseline, 6 months and 12 months |
|  |  | Changes in Estimated Glomerular Filtration Rate (eGFR) | Change from baseline |  | Baseline, 6 months and 12 months |
|  |  | Urine albumin-to-creatinine ratio (ACR) | Change from baseline |  | Baseline, 6 months and 12 months |
|  |  | Changes in Glycemia | Change from baseline |  | Baseline, 6 months and 12 months |
|  | Description of blood pressure | | Value at baseline and final value | Mean ± SD | Baseline and 12 months |
|  | Description of body mass index (BMI) | | Value at baseline and final value | Mean ± SD | Baseline and 12 months |
|  | DIPAR tool perception and acceptability | Perceived patient and healthcare provider satisfaction and usefulness of the DIPAR tool | Through semi-structured interviews and focus group discussions | Qualitative data analyzed using thematic analysis | Assessed after 12 months post-implementation |
|  | DIPAR tool usability | Assessment of patient’s usability of the DIPAR tool | Scores derived from the mHealth App Usability Questionnaire; both English and Dutch versions | Data summarized from questionnaire with descriptive statistics | Assessed at 14 months post-implementation |
|  | Participant engagement with the consultation dashboard | Completion of the structured patient questionnaire within the MyNexuzhealth platform | Total number of questionnaire entries and number of unique participants completing at least one questionnaire | Descriptive statistics (counts, proportions, median/IQR where appropriate) | Throughout the 12-month follow-up |
| Study specific adverse events | Predefined study-specific adverse | - Technical issues or reliability - User challenges - Breaches in privacy or personal information | Reporting by participants in intervention group | Qualitative description and summary | Assessed after 12 months post-implementation |
